# Incels, autism, and hopelessness: affective incorporation of online interaction as a challenge for phenomenological psychopathology

**DOI:** 10.3389/fpsyg.2023.1235929

**Published:** 2023-12-06

**Authors:** Sanna K. Tirkkonen, Daniel Vespermann

**Affiliations:** ^1^Practical Philosophy, Faculty of Social Sciences, University of Helsinki, Helsinki, Finland; ^2^Section of Phenomenological Psychopathology and Psychotherapy, Department of General Psychiatry, Heidelberg University Hospital, Heidelberg, Germany

**Keywords:** incels, autism, hopelessness, body image, affective scaffolding, online affectivity, social connection, phenomenological psychopathology

## Abstract

Recent research has drawn attention to the prevalence of self-reported autism within online communities of involuntary celibates (incels). These studies suggest that some individuals with autism may be particularly vulnerable to the impact of incel forums and the hopelessness they generate. However, a more precise description of the experiential connection between inceldom, self-reported autism, and hopelessness has remained unarticulated. Therefore, this article combines empirical studies on the incel community with phenomenological and embodiment approaches to autism, hopelessness, and online affectivity. We analyze three interrelated aspects of online interactions in incel communities – worldview, bodily self-relation, and mutual dismissals – and examine how these elements contribute to the consolidation of the loss of significant life possibilities. By investigating the potential negative influence of specific online environments on affective dispositions, our approach contributes to the debate on current challenges to “situate” phenomenological psychopathology.

## 1 Introduction

Today, phenomenologists have become increasingly interested in investigating affectivity and social interaction in online environments (Fuchs, 2014; Krueger and Osler, 2019; Candiotto, 2022; Osler and Krueger, 2022). Whereas most phenomenological approaches have dealt with the similarities or dissimilarities between online and offline interactions, very few (Osler and Krueger, 2022; Fuchs, 2022b) have investigated the potential role of online interactions for psychopathological phenomena. To our knowledge, it has not yet been investigated in phenomenological psychopathology, how online interactions might influence neurodevelopmental conditions. Recently we have seen a rise in phenomenological approaches to autism that acknowledge how neurotypical social contexts negatively shape non-neurotypical formation of experience (Krueger, 2021b; Boldsen, 2022). However, the potential impact of online interactions has largely been understood as beneficial, whereas the possible negative effects have not yet been in the focus.

In this article, we focus on a current phenomenon that has attracted much attention both in academic research and online: using phenomenological means, we analyze self-reported autism and its association with hopelessness in the online communities of involuntary celibates (incels).

“Incel” refers to involuntary celibacy, but the term does not describe someone who is living in celibacy against their will. Rather, it is a self-proclaimed identity that involves accepting a set of beliefs about sexual attraction and gender roles in society. The set of beliefs originates from a network of anti-feminist online communities known as “the manosphere.” Manosphere communities promote a hierarchical understanding of gender relations fostered by specific terminology, pseudo-scientific theories, and categorizations of human beings based on their looks, wealth, and social status (Ging, 2019; Vallerga and Zurbriggen, 2022). Incels themselves emphasize that they do not form a uniform movement but differ in their problems, attitudes, political views, and life situations. According to studies and online surveys, however, they do have certain features in common: in a survey carried out in October 2019, 100% self-identify as male, and 95% believe in “the black pill” (Stijelja and Mishara, 2023, 722–723). “The black pill” refers to an outlook or “philosophy” according to which men who have certain disadvantages in the dating culture cannot do anything to improve their attractiveness, for example by changing their behavior or physical appearance. The black pill philosophy argues that physical attractiveness determines men’s dating success in Western societies in general (Incels Wiki, 2023), but in contrast to manosphere communities that offer self-improvement and pick-up strategies, incels have given up hope that their alleged disadvantages could be fixed in order to find an intimate partner.

When investigative journalist Naama Kates interviews in her *Incel* podcast a person (“K”), who self-identifies both as an incel and as someone on the autism spectrum,^1^ he questions the emphasis on looks and explains how he sees the causes of inceldom (Kates, 2021):

The majority of incels are incels because of mental conditions, not because of their looks. So, the big issue is this: let’s say you look under average, or average, and if you’re neuro-typical, if you don’t have autism, you’re able to get a girlfriend, just by, for example, talking with a girl, like wooing her in high school, it’s still possible, believe it or not […] but if you’re autistic, it’s completely over for you.

In the quote, the interviewee duly criticizes the idea that physical appearance alone determines whether one finds a romantic or sexual partner. However, there is also a sense of *hopelessness* in the quote when he suggests that with autism, there is even less one can do.

Recently, several studies have investigated the mental health aspects of self-avowed incels and noted the prevalence of autism in their self-reports (Broyd et al., 2022; Sparks et al., 2022, 2023; Speckhard and Ellenberg, 2022; Moskalenko et al., 2022a,b). There would appear to be specific links between autism and inceldom, and roughly a quarter of the users of incel forums have reported a formal diagnosis of autism (Hoffman et al., 2020; Speckhard and Ellenberg, 2022; Moskalenko et al., 2022b). In addition, research that focuses on incels in forensic psychiatry, radicalization and terrorism studies became interested in autism after noticing that many of the incels who committed violent attacks and mass shootings against women had a formal autism spectrum disorder (ASD) diagnosis (Broyd et al., 2022; Woodbury-Smith et al., 2022). Studies by Broyd et al. (2022) and Hoffman et al. (2020) mention 50 incel-related violent incidents since 2014. Due to mixed research results, however, it has been emphasized that one should be extremely cautious when making generalizations and associating violence with autism (Williams D. J. et al., 2021, 395–396). Autism alone does not make anyone an incel, which is a self-acclaimed cultural category, and violence, radicalization, and hostile attitudes toward women cannot be explained by autism.

It seems, however, that some autistic people may be particularly vulnerable to the impacts of the incel forums. Therefore, it has been suggested that it would be highly relevant to know whether the forums “have contributed to creating or exacerbating underlying vulnerabilities through frequent discussions of suicide and hopelessness” (Stijelja and Mishara, 2023, 728). Thus far, it has been more common to use statistical methods to investigate incels, either through the lens of mental health or sociopolitical radicalization. A more precise description of the *experiential* connection between inceldom, self-reported autism, and hopelessness has thus far remained unarticulated, even though incels’ first-person accounts make their experiences particularly suitable for phenomenological analysis.

In this article, we analyze these experiential connections by means of phenomenological psychopathology. Instead of focusing merely on the individual experience of autism in the incel community, we analyze the impacts of the online environments on a specific vulnerable condition. We are particularly interested in the aggravation of hopelessness in the forums. Hence, we explore the ways in which social interactions, and the online environment in which these interactions take place, lead to incels’ self-marginalization and exacerbation of their condition. To this end, our research combines empirical studies on incels’ self-reported autism with current advances in the phenomenology of autism, hopelessness, and online affectivity. We suggest that the specific social interactions significantly impact the self-understanding, bodily self-relation, and affective dispositions of persons already in a vulnerable position.

We proceed as follows: First, we provide a review of the current empirical findings on the links between inceldom and autism. Second, we explain how theoretical advances in phenomenological psychopathology may help us better understand how they are connected in experience. Third, we clarify and distinguish different forms of hopelessness by phenomenological means. We identify three factors that reinforce the collective deprivation of hope in incel forums (scientific determinism, body image dominance, and mutually dismissive communities) and show how their impacts exceed the boundaries of online environments. By drawing on 4E approaches to affectivity, we describe how the specific worldview, bodily self-relation, and interactional features of incel communities become incorporated. We argue that these specific types of online interaction, with their testimonial immunization, procedural integration, and ultimately mind-invading potentials, transform and consolidate affective dispositions. We suggest that this affective incorporation of hopelessness exacerbates pre-existing difficulties in establishing social connections and reinforces social marginalization. Finally, we argue that our approach contributes to the debate on the current challenges of “situating” phenomenological psychopathology.

## 2 Incels’ self-reported autism, violence, and hopelessness

In the podcast series *Incel* (Kates, 2021), an interviewee (“K”) describes his own situation and the connection between inceldom and autism as follows:

It was impossible for me to get a connection with girls and women. At that time, I didn’t know why that was. And then I always tried to find a girlfriend, and I told myself if I don’t find a girlfriend when I’m 18, I’m gonna kill myself because I’m losing, I’m failing. Everybody around me has a girlfriend. Everyone around me is having sex. […]

I lost a ton of weight, I improved my looks, and I tried to do that, but the issue is that all of that was for nothing. You know why? Because, as it turned out, I have autism. I’m on the autistic spectrum. And this caused me so much pain and trouble during high school, during college, and that is the main reason why I am an incel.

This passage describes from the first-person perspective how late-*diagnosed* autism is perceived as the main explanatory factor for the inability to form intimate relationships. It also captures the psychological distress the person is going through: suicidal thoughts, feelings of failure in a highly normative social setting, and grief at losing the type of life one had envisioned for oneself.

Even though empirical studies still often refer to autism spectrum *disorder* (ASD), according to the standard definition, autism is not a mental disorder but a neurodevelopmental condition that typically manifests in early childhood. As we are dealing with a spectrum, the autistic traits vary in their severity and quality.^2^ They often involve difficulties in social interaction and communication and may include repetitive patterns of behavior, a preference for routine and predictability, and sometimes intense special interests (White et al., 2017; APA Dictionary of Psychology, 2023). One of the autism diagnostic scales (RAADS-14 Screen), for example, specifies that autism often entails certain social anxieties (e.g., difficulties in functioning in social situations, making friends), mentalizing deficits (e.g., difficulty interpreting the feelings and gestures of others), and sensory reactivity (e.g., a feeling of sensory overload and a need to isolate oneself to shut down the senses) (Eriksson et al., 2013; McLeod and Anderson, 2023, p. 3480). Phenomenological psychopathology also has a tradition of investigating autism in the context of schizophrenia (see Parnas and Bovet, 1991; Stanghellini and Ballerini, 2011; Froese et al., 2013; Palumbo et al., 2021), but here we refer to the self-reported autistic traits and formal diagnoses identified in the empirical research literature on incels.

Studies that have surveyed incels’ mental health have noted that self-reported autism stands out in their data (Speckhard and Ellenberg, 2022; Moskalenko et al., 2022b). In addition to ASD, depression and anxiety are significantly higher amongst the incel participants compared to the American adult population. Autism is also a frequently debated topic in the incel forums. Autism spectrum tests are circulated, which might itself enhance the identification of certain difficulties typical of autism.

Studies thus make a clear distinction between *self-reported* psychological problems and formal diagnoses made by healthcare professionals. For example, Moskalenko et al. (2022b) surveyed the mental health of 278 participants who self-identified as incels. Of their participants, 95% (261) reported experiencing depression, 94% (257) anxiety, and 74% (199) some autism spectrum traits. When the participants were asked about a formal diagnosis, 37% (102) reported depression, 37% (101) anxiety, and 18% (50) autism spectrum disorder. Additionally, 56% reported that they had never been diagnosed. Similarly, Speckhard and Ellenberg (2022) investigated respondents’ self-reported mental health challenges, including the perceived symptoms of different mental disorders, addictions, self-harm, and suicidal ideation. In their study, 64.3% of participants reported that they had experienced depressive symptoms, 59.6% anxiety, and 24.6% traits of autism spectrum disorder. In addition, 47.8% reported the presence of suicidal ideations. An online survey carried out by an Incels.co moderator, in turn, reported an even higher number of suicidal thoughts: 67.5% of the participants considered suicide a serious option if they could not find any improvement in their life situation (Stijelja and Mishara, 2023). In the survey, 59% of participants reported depression, 74% stress and anxiety, and 25% autism.

In light of these studies and surveys, the reported rate of formal ASD diagnosis is roughly 20%, but the number of self-reported symptoms varies depending on the ways in which the questions have been formulated. However, compared to the global prevalence of autism spectrum disorder (0.62%), the reported formal diagnosis of autism among incels is remarkably high (Speckhard and Ellenberg, 2022). The rate of ASD diagnosis among incels also clearly contrasts with the estimate that 3.6% of adult males in the U.S. are on the spectrum (Moskalenko et al., 2022b). Furthermore, autism often goes undiagnosed: some studies estimate that there are 25% more cases of ASD than diagnosed cases (*ibid.*; Wiggins et al., 2020).

Several studies have also addressed the question of the extent to which the violent behavior of certain incels and autism can be associated (Speckhard et al., 2021; Broyd et al., 2022; Speckhard and Ellenberg, 2022; Woodbury-Smith et al., 2022; Moskalenko et al., 2022a,b). Some studies argue that perceiving incels as violent and misogynistic terrorists only increases their stigmatization, even though the majority of incels do not exhibit violent traits (Moskalenko et al., 2022a,b). They also note that the majority of incels (52%) consider themselves less violent than they are perceived to be (*ibid.*). However, based on the statistics, the radicalized minority of incels are *more likely* to be on the neuro-divergent spectrum with a formal diagnosis (Moskalenko et al., 2022b). A study that surveyed the experienced effects of online incel forums shows that the “self-reported intensity of autism spectrum traits was significantly associated with agreement that the forum has made the respondent feel violent […] and misogynistic (Speckhard and Ellenberg, 2022).” A minority of incels do embrace violence, glorify incel killers, fantasize about rape and belittle its consequences (Moskalenko et al., 2022a,b). These attitudes, emotional responses, and patterns of thought are intensified in the forums. Accordingly, the forums may function as negative echo chambers, particularly for those who already have difficulties in communicating with others (Speckhard and Ellenberg, 2022). It would therefore appear that certain vulnerabilities in autism spectrum traits may increase the risk of radicalization in the context of online communication (Williams D. J. et al., 2021; Williams G. L. et al., 2021; Broyd et al., 2022). In addition to difficulties in social interaction, these vulnerabilities include a tendency toward literal thinking and interpretation (Broyd et al., 2022; Woodbury-Smith et al., 2022). Importantly, violent incels on the spectrum also exhibit other risk factors – most commonly a history of being bullied (Moskalenko et al., 2022b), suggesting that it is not enough to treat autism merely as an individual problem, but to focus on the social interactions and environments in which people are trying to navigate.

That said, hopelessness is also mentioned in several studies as one of the key risk factors for violent behavior (van Brunt et al., 2021; Broyd et al., 2022; Speckhard and Ellenberg, 2022; Woodbury-Smith et al., 2022). Even if there is not necessarily a link between hopelessness and violence, it is worrisome that in the survey by Speckhard et al. (2021), the majority of incels (54.4%) reported that these specific online forums make them feel hopeless. In general, hopelessness is highlighted in the first-person narratives of autistic people who struggle with interpersonal dynamics but observe that socially skillful individuals are always the popular ones (Woodbury-Smith et al., 2022, 3). When Speckhard & Ellenberg asked their incel participants whether they would date themselves, some of those who replied negatively referred explicitly to their autism: “I’m too autistic for any normal person to even relate to,” or “I’m just a regular guy who is socially autistic and sub-human looking” (Speckhard and Ellenberg, 2022). Similarly to the quote above, the possibility of ever having intimate relationships is characterized as being “over” for those on the spectrum, and adopting the black pill ideology only deepens these feelings of hopelessness.

## 3 Phenomenological approaches to autism: bodily attunement and situatedness

Thus far, we have referred to the statistics in previous research on the prevalence of autism in the incel community. However, to better understand the link between inceldom and autism from an experiential point of view, it is useful to introduce the current advances in the phenomenological psychopathology of autism spectrum conditions (ASC) into the discussion.

Most importantly, phenomenological approaches to autism have challenged the prevailing ways of defining autism as a deficiency of cognitive functioning – as a failure to understand the mental states of others and oneself (Fuchs, 2015; Krueger, 2021b; Boldsen, 2022, 2). Instead, phenomenologists have argued that difficulties in interacting with neurotypical others stem from a different way of being bodily attuned to the environment and the expressive gestures of others (Fuchs, 2015; Bizzari, 2018; Krueger, 2021b; Bader and Fuchs, 2022; Boldsen, 2022). This conceptual change is sometimes called “an embodied turn” in autism research (Boldsen, 2022). The idea is that in neurotypical experience, the meaning of others’ gestures is implicitly and immediately present in social interaction, without having to first consider what a handshake, eye contact, a smile, a raised eyebrow, leaning forward, or turning away could possibly signify in a particular situation. Of course, it is always possible for anyone to misinterpret others and the situation and to feel confused, but in neurotypical experience the reflective activity often sets in afterwards, if at all. In other words, the differences between neurotypical and autistic experiences of interaction take place at the prereflective level (*ibid.*). Oren Bader and Thomas Fuchs have argued, for example, that those with ASC have difficulties in *gestalt perception*, which unites the gestures, attitudes, and behaviors of others into a meaningful whole in light of the overall situation (Bader and Fuchs, 2022).

For these reasons, it might also be difficult for incels on the spectrum to recognize just what has “gone wrong” in their attempts to approach others, especially potential intimate partners. Neurotypical people, in turn, may find the bodily gestures and rhythms of conversation off-tune and awkward compared to what they are used to, and the sometimes blunt and honest answers might be taken as offensive (Krueger, 2021b, 29). From an autistic point of view, by contrast, interactions may in general lack corporeal familiarity and appear chaotic, unpredictable, and intrusive (Boldsen, 2022). Therefore, it is not unusual for people on the autism spectrum to go through deliberate, laborious cognitive processes in social situations to find social meaning (*ibid.*, 9). High-functioning autistic people employ cognitive strategies to compensate for their difficulties in adapting to neurotypical ways of being attuned to the gestures of other people (Zahavi and Parnas, 2003; Froese et al., 2013; Fuchs, 2015, 198). Incels on the spectrum often recognize that they struggle to engage in neurotypical patterns of interaction, and therefore seek ways to make sense of its implicit rules and try to explain how intimate relationships are formed. The black pill theory, with its categorizations and rankings of attractiveness and social desirability, can thus be understood as one of the strategic means of trying to grasp and organize the social cues implicit in neurotypical behavior. In other words, it may function as a tool for organizing a social world that otherwise appears random and unpredictable (Kates, 2021).

In addition to reconceptualizing ASC by analyzing the underlying bodily (un)familiarity of an individual’s social perception, phenomenological studies on autism have also emphasized the situatedness of human experience – the ways in which one finds oneself in the world with others. This has included pointing out that social spaces and their interactions are usually organized according to neurotypical behavior (Krueger, 2021b; Bader and Fuchs, 2022; Boldsen, 2022). Thus, the non-neurotypical difficulties stem from a different style of being-in-the-world, which is actively discouraged or even sanctioned by others. For a person with autism, neurotypical environments may appear inhibiting, threatening, and oppressive.

Joel Krueger, for example, has argued in the context of autism that the lived space is not just a physical place that bodies reside in, but a space in which bodies and their possible ways of being are formed in relation to others (Krueger, 2021b, 23). This in-betweenness has a normative quality, as Krueger puts it: “[the shared spaces of betweenness] take shape around the values, practices, and preferences of those who inhabit them. Considering these normative dimensions, some bodies feel more at home in – or are allowed to feel more at home in – our shared spaces than are others (*ibid.*).” Referring to Ahmed (2006), he goes on to say that in our everyday lives we actively navigate in and through these relations and try to “find our way” in them without giving much thought to it. When one does pay attention to the social character and conditions of this navigation, one has usually already lost access to it, or, perhaps for some, it has never been truly self-evident. Krueger and Ahmed call this loss or absence of access to the shared social space *disorientation*: it can take different forms and intensities, but it refers to a felt sense of *not finding one’s way* in social interaction (*ibid.*; see Ahmed, 2006).

In the case of incels, it is useful to distinguish between two forms of disorientation: epistemic, and bodily-affective (Krueger, 2021b). The latter, *bodily-affective disorientation*, refers to the above-described difficulties of being bodily attuned to neurotypical others, fitting into their world, and identifying the emotions that this dis-attunement may involve. *Epistemic disorientation*, in turn, refers to a major shift in one’s core beliefs, which can have a significant effect on the way in which one finds oneself in the world, and interprets social interactions and one’s relationship with others (Krueger, 2021b, 24). Typical examples of epistemic disorientation could be losing religious faith or finding it, both of which can impact a person’s daily behavior, interactions, and the ways in which the surrounding world is perceived as meaningful or meaningless. Similarly, becoming “black-pilled” is characterized by incels as a revelation of truth about the “facts of life,” women and their preferences, gender relations, and how intimate relationships are formed. After arriving at this newfound enlightenment about “how things really are,” the former belief system seems “delusional” and uninformed, and it appears to benefit only those with the right physical attributes, status, and power to find sexual and intimate partners. Krueger argues that bodily-affective and epistemic disorientation do not necessarily occur together, but in the case of incels the distinction between them is not clear-cut. Even if adopting the black-pill philosophy could be characterized as an epistemic change (presented as rational and scientific) in one’s former familiarity and taken-for-grantedness, it clearly involves affective dimensions, ranging from hopelessness, disappointment, and sorrow to antagonistic emotions such as anger, hatred, misogyny, and *ressentiment* (Tietjen and Tirkkonen, 2023).

As phenomenological studies on autism understand neurotypical environments as normative spaces that inhibit the non-neurotypical ways of being, they have stressed the role of online environments in creating spaces with new norms, styles of expression, and vocabularies (Hacking, 2009; Krueger, 2021b, 30). Online environments are thus presented in a positive light as spaces of mutual recognition and acceptance, where non-neurotypical persons can also feel comfortable and at home. However, what is still lacking in phenomenological studies on autism is a critical analysis of the negative effects of online environments on one’s self-perception and felt possibilities of forming relationships. Given the above-presented ratio of autistic people within incel communities, we analyze *how* these online interactions may have such a negative impact. In the next section, we first describe how empirical studies have stressed the crucial role of hopelessness in these communities, before analyzing hopelessness through an existential phenomenological lens.

## 4 Intentional and radical hopelessness

Several empirical studies on incels mention hopelessness as a significant dimension of their experience (Williams and Arntfield, 2020; Speckhard et al., 2021; Sparks et al., 2022; Stijelja and Mishara, 2023). The incel vocabulary sometimes differentiates between three strategies for living through the pain of not being able to form intimate relationships: “hope,” “cope,” and “rope” (Sparks et al., 2022). In general, the black-pill philosophy rejects a hopeful outlook on life, particularly any advice that aims at self-improvement or individual healing. When it comes to intimate relationships, incels engage in highly self-critical rumination (Sparks et al., 2022). Moreover, those who simply try to accept their situation without challenging the prevalent social norms are easily labeled as “copes,” which has a pejorative meaning. “Rope” in turn refers to suicide. A number of studies have investigated the posts on suicide in the forums and noted that suicide ideations also encourage others to carry them out in practice (Daly and Laskovtsov, 2021; Laplante and Boislard, 2021; Sparks et al., 2022). The rope posts are characterized by overall hopelessness in terms of improving one’s life situation or finding a romantic or sexual partner (Laplante and Boislard, 2021; Sparks et al., 2022). Furthermore, a study by Williams and Arntfield (2020), focusing exclusively on homicidal incels, has shown that they not only experience hopelessness in one sphere of life, in intimate relationships, but in multiple areas of their everyday lives, including family relations, employment, and social connections in general (Williams and Arntfield, 2020, 37). The study also identifies hopelessness in body perception issues and psychological distress. It is often the case that incels who are prone to violence have also suffered from significant losses (*ibid.*, 41).

In sum, the studies have shown that hopelessness is not restricted to one specific dimension of everyday experience – rather, it is a more encompassing affective state that extends to different spheres of life.

However, the aim of the empirical studies is not to ask how hopelessness should be defined or understood in the first place, and they do not provide *phenomenological descriptions* of how feelings of hopelessness have a bearing on experience. In order to understand the types of hopelessness that “the black pill” involves, we must first grasp what it means to lose hope in the first place, and consequently what is meant by “hope.” This is where phenomenological studies are more than helpful.

First of all, we can make a distinction between two different types of hope: intentional and pre-intentional (or “radical”). Despite the increasing philosophical interest in hope, most work on the topic deals with what might be called “intentional hope,” hope *for something* (Bovens, 1999; Meirav, 2009; for an overview, see Rioux, 2021). Taking the incel cases as a guide, the corresponding intentional objects might vary in their determinacy but, all in all, whether one hopes to have a girlfriend or to have sex, there is some state of affairs that one hopes will occur. In the research literature, intentional hope is commonly defined by some variation of the so-called “standard account,” according to which hope is understood as a combination of the desirability of *p* and a belief in some degree of the probability of *p* (Meirav, 2009).

Yet some authors claim that there is also a more fundamental capacity for hope: a “radical” (Lear, 2006) or “pre-intentional hope” (Ratcliffe, 2013). The different variants of fundamental hope imply a sense of having possibilities at all. In other words, rather than representing probabilistic beliefs or giving credence to more or less certain occurrences, fundamental hope amounts instead to a felt sense of possibility. This raises the question of what to hope for or rather *how* to hope if there is no sufficiently circumscribed intentional object of hope.

One way to understand this is to see fundamental hope as a prerequisite for intentional hopes. For example, hoping to have a girlfriend presupposes a basic sense that the future actually holds anything positive at all. Thus, the desirability of the outcome in fundamental hope implies a wish for personal or collective flourishing. This assumes a tight connection between fundamental hope and agency in that the possibilities opened up by fundamental hope reflect the potential realization of one’s commitments, projects, or values as they constitute one’s personal identity. Consequently, hoping for something specific to occur already presupposes having a sense that what holds personal significance can be realizable in the first place. Yet, as will become clear below, the aforementioned should not be understood by way of a strict dichotomy between intentional and pre-intentional hope. Rather, we also find cases of (loss of) hope more profound than the hope that a certain event happens, and less fundamental than having a sense of agential possibilities at all. To clarify this, it is helpful to take a closer look at what it means to lose hope.

To begin with, the loss of intentional hope that something will happen is a rather mundane event that does not impair our general sense of meaningful possibilities. Yet something akin to the latter might happen. According to Ratcliffe, such a fundamental loss of hope could be divided into two broad categories. The first comprises a loss of all intentional hopes, which is compatible with Lear’s (2006) “radical hope,” the hope that “something good will come.” The second category refers to an even more profound loss of hope in which we “also [lose] an orientation that is presupposed by the possibility of hoping for anything” (Ratcliffe, 2013, 604).

The latter type of loss is still open to further qualitative distinctions. Ratcliffe characterizes the deepest form of loss as the “absence of the capacity to hope for anything combined with lack of awareness that anything has been lost” (2013, 605). According to Ratcliffe, this type of hopelessness might, for example, be “found in severe cases of depression” (*ibid.*). Other, less severe forms concern “loss of ‘aspiring hope’ (hope of bettering oneself or improving one’s life),” a “loss of one’s own future as a dimension of hope” and a “loss of trust in the world, which renders all hope ‘fragile’ and may restrict the scope of potential hope contents” (*ibid.*). What these forms of losing hope have in common is that they deprive the future of significant possibilities.

Returning to the sphere of romantic relationships, we can picture the loss of hope of finding a date for the weekend, which might concern both private and social life, but does not necessarily disturb the anticipation of having other meaningful possibilities in life. Losing “radical hope,” by contrast, will completely deprive all plans, intentions, and interactions of their meaning. If the whole system of significance has vanished, no particular hopes make any sense anymore. To clarify the extent to which incels experience such profound hopelessness, we focus on the characteristic type of loss: For incels, there has never been a particular (romantic) significant other, yet they do express desperation and grief over a specific loss.

We suggest that this type of grieving has to do with a “non-death loss,” which refers to cases of mourning the absence of something or someone significant in one’s life without it being identical to bereavement (Richardson and Millar, 2022). It can be experienced in the context of life changes, but also, we claim, when realizing that an assumed or long-wished-for life scenario will not take place. Incel studies have paid attention to the experience of missing a developmental milestone by not having sex at a certain age, which exacerbates the feeling of being different from one’s peers (Donnelly et al., 2001; Stijelja and Mishara, 2023).

Crucially, these losses and the emotional pain they imply are often conditioned on societal expectations and result from internalized *cultural life scripts* (Habermas and Bluck, 2000; Berntsen and Rubin, 2004). Cultural life scripts denote a set of life events that are expected to take place and be experienced in the course of a “normal” life. In other words, “not having sex” with a partner implies a whole set of cultural norms on how life is supposed to proceed. Importantly, as these norms are sociocultural in origin, not feeling capable of completing these “milestones” will be experienced as having consequences for social connections in general. In societies in which marriage and having a family are understood as default steps in one’s life, failing to find a partner might feel like, and to some extent actually amount to, social isolation: one is essentially excluded from numerous social opportunities in which having a partner or children is a tacit or factual requirement. In feeling desynchronized with regard to a certain cultural life script, we might feel out of sync with others in general and thus deprived of a conventional set of social possibilities.

How should we understand the grief over the loss that many incels are experiencing? As Ratcliffe (2013, 602) puts it: “Grief is experienced as a catastrophe that befalls one’s whole world.” Now, ideally, grief is only a transitory state that may be overcome after a period of time. Yet grieving can also acquire a trait-like quality, particularly when lacking social support. Accordingly, the “catastrophic” state becomes permanent, and even particular hopes that may have persisted at the onset of grieving are lost. In the incel community, the once-lived hope of having a romantic or sexual partner is turned into a permanent loss – an identity of being excluded from society and deprived of recognition by others due to not conforming to the presupposed norms of masculinity.

Where can we now locate this experience of non-death loss among the different types of hopelessness? Given the distinctions just presented, we can see that “black-pill hopelessness,” and in particular the “rope option,” is compatible with some type of *fundamental hopelessness*. Taking into account that incels are highly aware of what has been lost, their hopelessness seems to be captured best by what Ratcliffe (2013, 605) has termed “loss of aspiring hope” and “demoralization.” The former describes a loss of “hope for bettering oneself or improving one’s life” (*ibid.*), whereas the latter amounts to “a loss of a sense of one’s future as a dimension of hope” (*ibid.*). Both variants differ from full-blown depression, and demoralization does not even have to exclude the “capacity to hope” entirely (*ibid.*). As we will clarify later, this capacity might be intact in regard to hoping to belong to a community of equals (see section 5.3 below). Still, both types of hopelessness are fundamental enough to count as severe deprivation of one’s overall sense of meaningful possibilities.

In the following section, we will examine how incel communities consolidate and ultimately exacerbate this hopelessness.

## 5 Dimensions of “black pill” hopelessness *–* exacerbating social disconnection

How does “taking the black pill” *–* becoming aware of an irrevocable loss of ever having a fulfilled romantic partnership *–* actually come about? Or, put differently, how does the non-death loss of a certain type of social connection become consolidated into a permanent state of hopelessness? In the following section, we analyze the pivotal aspects of online interactions in incel communities and how they perpetuate the loss of significant life possibilities. To this end, we look at three interrelated dimensions: worldview, bodily self-relation, and intersubjective communication styles.

In line with recent ideas on “phenomenological causality” (Wilde, 2022), we consider the interrelations between these dimensions to exhibit implicatory (Sass, 2010) or “motivational” relations (Fuchs, 2022a). In short, implicatory or motivational relations are taken to be stronger than correlations and, at the same time, might include volitional aspects, such as compensatory coping strategies related to more basic alterations in experience. While we cannot argue for the universal validity of these experiential interrelations, we espouse an interdependency in the specific case we are investigating. Specifically, we claim that the particular manifestations of each of the three dimensions tap into common vulnerabilities in autism and together consolidate hopelessness.

### 5.1 Scientistic determinism as a hallmark of black pill ideology

Several authors (Bosticco and Thompson, 2005; Nelson et al., 2022; Ratcliffe and Byrne, 2022) have established that narrative interactions are crucial to alleviate the distress and feeling of loss in grief. By narrating, we eventually try to reinvigorate personally significant possibilities, in short, to regain hope. However, the incel communities endorse quite a different type of sense-making.

The incel community knows two ways of “taking the black pill” – philosophically and scientifically. Whereas the former amounts to a general attitude of hopelessness based on “lookism,” taking physical attractiveness as the decisive parameter for finding a sexual partner, the latter delves into scientific data supposedly backing up the rather speculative ideas of the general black pill philosophy. However, both types of “blackpilling” comprise the idea of biological determinism, either of a more folk-theoretical or natural scientific blend. In the “Scientific Blackpill” (Incels Wiki, 2023), evolutionary explanations, causal mechanisms, statistical “evidence,” and fine-grained taxonomies abound and lend allegedly neutral scientific support to the perceived asymmetrical sexual power relations between women and men. We suggest that as an exclusive means of self-understanding, biological reductionist and particularly deterministic explanations facilitate hopelessness.

To begin with, reductionist explanations already imply an epistemic standpoint that is hard to reconcile with individual agency. To be sure, there is no clear consensus on what makes a causal explanation properly reductionist and when it is compatible with teleological or even narrative elements (Keil, 2006; Beatty, 2017; Aronowitz and Lombrozo, 2020). However, a rather uncontroversial perspective takes reductive causal explanations of behavior as subpersonal, focusing on abstract properties of and relations between defined entities and relinquishing motivational aspects (Aronowitz and Lombrozo, 2020). Similarly, when narrating we preselect the material and thus abstract from details. Unlike mechanistic explanations, however, we “organize” and “color” (Goldie, 2014, 11) it from “a certain perspective or perspectives,” giving “coherence, meaningfulness, and evaluative and emotional import” (*ibid.*, 2) to what is narrated. These latter attributes are diametrically opposed to the epistemic values of reductionist research programs in natural sciences. To summarize, in reductive explanations, nothing appeals to individual concerns, projects, or values.

According to “scientific blackpilling,” psychological processes are exclusively governed by evolutionary functions deprived of other motivational factors than biologically defined self-interest. Ultimately, these functions are exhaustively determined by genetic dispositions, and any potential romantic encounter thus becomes a mere phenotypical expression contingent upon these dispositions. At the personal level, subjects have only those options available to them that have been assigned to them by the genetically predetermined “sexual market value” (SMV). In this sense, it is the deterministic and reductive character of the explanations sought for their situations that facilitates hopelessness.

It makes a crucial difference whether one makes sense of one’s life – in this case (the lack of) romantic relations with others – through biological reductive explanations or narrative understanding. Even without endorsing determinism, a detached third-person perspective on highly intimate aspects of one’s life will diminish the feeling that one can be a locus of agency in a relevant sense. In fact, the latter is precisely what “blackpillers” deride in those who try to improve their situation and work around the biological facts.

The way we make sense of our situation extends to our affective dispositions. This becomes clear when we consider the role of narrative sense-making for emotion regulation (Pennebaker and Seagal, 1999; Angus and Greenberg, 2011). Even without an explicit regulative purpose, conceptual aspects factor into affective dispositions (Slaby and Stephan, 2008), including fundamental hope. Thus, it stands to reason that continuous endorsement of a particular conceptual framework, such as “narrative-agential” or “causal-deterministic,” also influences our affective states.

When explaining one’s life situation by way of deterministic reasoning, we may expect that such (self-)explanations involve feelings of futility, or, for that matter, hopelessness. If a course of events is predetermined, no intervention will be possible, and consequently all intentions formed are only effective inasmuch as they serve to execute irrevocable laws, subpersonal mechanisms, and so forth (Holton, 2009). The belief in diminished self-efficacy and thus one’s agential capabilities will naturally restrict the scope of one’s felt possibilities. To be fair, taking the black pill might not amount to a global fatalism leaving one deprived of possibilities in every respect. Yet the significant possibilities, as they would spring from a felt agency in regard to romantic and intimate relationships, are exclusively subject to genetically determined evolutionary mechanisms. In contrast, a pivotal function of narratives is to connect past experiences with one’s present perspective, and thus to delineate future possibilities in a personally meaningful way (Hardt, 2018).

#### 5.1.1 Diminishing hope by reinforcing determinism in autistic people

As stated at the beginning of section 5, we assume motivational interrelations between the different experiential dimensions. In this subsection, we suggest that a tendency toward deterministic self-understanding may express a compensatory coping strategy related to specific cognitive traits common to many people with autism. In other words, a rather deterministic self-understanding might be implied in difficulties in establishing narrative continuity.

As noted above, the disorientation resulting from taking the “black pill” amounts to a particularly detached perspective on how one’s future life will unfold. It now appears striking that future thinking which relates to vivid self-identification seems comparatively difficult for many autistic people to accomplish (Lind et al., 2014). Since this identification with one’s future self is crucial for significant life possibilities, corresponding difficulties will also affect one’s capacity for hoping. In particular, recent findings suggest that autistic people often have difficulties with episodic future thinking, or future-oriented mental time travel (Feller et al., 2021; Ye et al., 2023). Inasmuch as impairments of episodic future thinking have been linked to depression (Hallford et al., 2018), a close connection with fundamental hopelessness seems likely. In line with this, findings on comparatively high rates of depression among autistic people (Hudson et al., 2019) might be linked to similar attentional biases and tendencies for repetitive cognition (Unruh et al., 2020). Repetitive cognition or rumination, in turn, correlates with less vivid and positive episodic future thinking (Anderson and Evans, 2015; Beaty et al., 2019).

As stated in the previous section, narrative sense-making provides a temporal and meaningful continuity of past experiences and possible personal futures. In addition to the reported difficulties with episodic future thinking, studies on self-identification in autobiographical memories suggest that autistic people also tend to struggle to identify emotionally with themselves in recalled episodes (Lind, 2010; Chaput et al., 2013; Lind et al., 2014). This lends additional weight to problems with narrative sense-making. When the duplication of perspectives – one’s remembered self and the remembering self – cannot be bridged by narrative sense-making, it is difficult to establish continuity between remembered episodes and current concerns. This, in turn, confers a compensatory function on third-person perspective-taking (Arnaud, 2020, 366). In other words, it has been suggested that in autism, one’s self-relation is often dominated by a third-person perspective (*ibid.*).

If we acknowledge that a third-person perspective toward one’s past and future implies a rather general perspective, with less emotional import, this seems to dovetail with rather mechanistic or even deterministic self-explanations. Recent findings (Berent et al., 2022) indeed suggest that those on the autism spectrum often lean toward physicalist and nativist ideas, both of which, admittedly, are not identical to determinism, yet show significant overlap and, in fact, often coincide. If it is true that autistic people tend toward third-person self-consciousness, and that this is moreover molded by deterministic ideas that restrict the horizon of (significant) possibilities, it can be assumed that the pervasive self-objectification in “blackpilling” further diminishes the felt sense of agency in interacting with others. To further substantiate this claim, in the next section we outline how body image dominance might hamper interoceptive abilities.

### 5.2 Self-reification: body image dominance in incel communities

Another aspect, closely related to the presumed subordination under evolutionary mechanisms, concerns (self-)reification through body image evaluations. In incel communities, outward appearance becomes by far the dominant factor in being able to form romantic relationships. What constitutes so-called “sexual market value” (SMV) is exclusively conditioned by physiognomic features. Correspondingly, many of the studies drawn on in “blackpilling” examine the role of specific bodily features for finding partners to procreate with. The underlying tendency to operationalize and predict mating success with physiognomic features consequently amounts to a decimal ranking system of sexual attractiveness. Some community members share photos of themselves to be assessed and classified by other incels, thereby enacting and reproducing the prevalent SMV ideology. Eventually, this contributes to shaping the bodily self through a third-person perspective on one’s own body.

None of the above is, of course, exclusive to incel communities. In fact, the rise of social media has been connected to an increase in body image dissatisfaction, eating disorders, and anxiety (Vannucci et al., 2017; Shensa et al., 2018; Jiotsa et al., 2021; Dane and Bhatia, 2023). Yet, embedded in a ranking system based on “scientific facts,” self-reification by body image dominance acquires a different quality. It is the inevitability of evolutionary laws which confers on body image the character of fate rather than malleability. Furthermore, the lack of social relationships outside of online interactions might lead to body image evaluations being disproportionately important for one’s bodily self-relation. Finally, the particular quality and homogeneity of body image assessments distinguishes incel community interactions from more mainstream social media: it is part and parcel of the self-identification processes of incels to be dismissive of one’s physical appearance (see section 5.3). Simultaneously, it becomes clear how a negative body image may involve a loss of significant possibilities: If one’s self-perception is grounded in a highly negative and ultimately unattractive body image, this will reduce the conceivability of scenarios in which one could be a romantic partner for someone and severely restrict the range of particular (intentional) hopes.

Furthermore, a negative body image might at least indirectly yield a more fundamental loss of hope. For incels, the whole realm of romantic possibilities is governed by the dominant role of outward appearance for finding romantic partners and a definite arithmetic calculus of how persons of different ranks will be able to find them. Under the unfavorable circumstances of a low rank, this will amount to a perceived impossibility of ever having a romantic relationship. However, as specified above, this felt impossibility implies a broader loss of social connections. In that sense, the absolute value of one’s SMV is tantamount to an irreversible and complete loss of significant possibilities of a certain type. As such, it is the commanding role of body image for self-understanding that massively constrains the role of significant possibilities.

Even if more profound forms of hopelessness (see section 4 above) are by definition based on a *felt* loss of significant possibilities, we can assume that once a certain body image has been adopted and has become a part of one’s identity, this also affects the implicit bodily orientation in one’s environment (*body schema*) (Pitron et al., 2018; Riva and Gaudio, 2018; Ataria and Tanaka, 2020). Accordingly, the range of significant possibilities is then not only affected by the inability to imagine possible scenarios but also constrained “bottom-up,” that is, by felt difficulties of bodily attuning. This raises the questions, which “felt dimension” of bodily attunement are we actually talking about and how can it be influenced by body image?.

#### 5.2.1 Diminishing hope for social connections by thwarting interoceptive abilities

As a working definition, *body image* refers to a set of “perceptions, beliefs, and attitudes” (Gallagher, 2005, 25) toward one’s body, that is, from a third-person perspective. However, “third-person perspective” or “image” does not necessarily imply a detached, “disembodied” relation to one’s own body. Hence we concur with non-representational usages of “body image” (Gallagher, 2021). The explicit self-relation via *body image* is intimately connected with the *body schema* as an implicit sensorimotor orientation.^3^ Despite this interrelation, we agree with Gallagher (2021) that we can single out an explicit way of relating to one’s body, for instance in referring to one’s body via quantitative measures like weight or height, as seen in the mirror, or according to socioculturally transmitted, often stereotypical body norms. As this list shows, body image as a way of relating to one’s body is open to include representations of one’s body as in pictures. These representations may become part of the body image as a phenomenological structure, for instance, as a habitual way of thinking about one’s body. Inasmuch as body image – as the body, as it appears to others – provides rich relational information (e.g., spatial, social), it also informs and may direct sensorimotor potentials, bodily comportment and postures (Pitron et al., 2018), as well as affective dimensions of bodily experience (Gaete and Fuchs, 2016).

Developmental psychological studies, again, suggest that processes related to body image and the felt dimension of bodily perception (“interoception”) interact in developing a bodily self (Dyrsdale and Tsakiris, 2021). As a consequence, this bodily self also emerges from evaluations implicit in the body image, such as social relations or size, that interact with arousal and motivational states resulting from the felt bodily dimension (Craig, 2009; Colombetti and Harrison, 2019). Feelings of fatigue, dizziness, or excitement influence how we project ourselves in the future and delineate possible future scenarios, and conversely, thinking of or seeing one’s body, for example in a medical setting, may induce feelings of illness, or a sudden ache, or at least guide our interpretations of these feelings (Leder, 2019, 314ff.). Below, we focus on empirical findings regarding conflicts between interoception and body image.

In general, relating to one’s own body predominantly via body image might have drastic consequences, as has been suggested, for instance, in regard to social media-inspired plastic surgery (Tremblay et al., 2021). Importantly, increased attention to body image has been linked to difficulties in interoceptive abilities (Tsakiris et al., 2011; Valenzuela-Moguillansky et al., 2017), which is part of the larger neurobiological and psychological construct of “interoception” (Quadt et al., 2018). Under this umbrella term, “interoceptive abilities” are part of the “psychological dimension” (*ibid.*) comprising interoceptive accuracy, sensibility, and awareness of bodily sensations like feeling hungry or being attentive to one’s heartbeat. Importantly, interoceptive abilities are crucial for the perception and interpretation of affective states, and deficiencies are assumed to be relevant for alexithymia. More precisely, lower interoceptive accuracy has been found to correlate with body dissatisfaction, self-objectification, and body image disturbances (Emanuelsen et al., 2015; Badou and Tsakiris, 2017; Zamariola et al., 2017), while lower interoceptive awareness and sensibility correlate with alexithymia (Zamariola et al., 2018; Scarpazza et al., 2022). For our current purpose, it is important to note that interoceptive abilities have been shown to be a determinant in facilitating social connections (Arnold et al., 2019; Baiano et al., 2021). In sum, interoceptive abilities differentially affect how we relate to our bodies, understand our emotions, and are able to foster social connections.

Notably, findings in autism research (Garfinkel et al., 2016; Proff et al., 2021) suggest that it is common in autism to have difficulties in sensing and interpreting bodily signals (e.g., whether one is hungry or why one’s heart is racing) (Quadt et al., 2018). Yet recent studies have questioned a direct correlation between interoceptive difficulties and autism, pointing instead to the mediating role of alexithymia, which nonetheless has a relatively high prevalence among autistic people (Shah et al., 2016; Kinnaird et al., 2019; Vaiouli and Panayiotou, 2021). For the current aim of examining how negative body images in incel communities might consolidate hopelessness, this dissociation of interoceptive difficulties and autism does not have to pose a problem. Since our focus is on those who struggle to establish social connections, we are ready to admit that this largely pertains to those who also have difficulties in understanding their emotions. Moreover, the fact that alexithymia does not seem to stem from difficulties in directly sensing bodily signals (Nicholson et al., 2018) leads us to the converse question of whether “higher-order” processes related to body image may have an influence on interoceptive awareness.

Since we are interested in asking how certain interaction patterns and ways of self-understanding might contribute to lasting hopelessness, we now ask: Does a continuous negative body image affect the way one is aware of one’s body? By stipulation, this would be tantamount to a literal *incorporation* of hopelessness. The available evidence is ambiguous, yet warrants starting from the basic assumption that there is an “antagonism” between weighing interoceptive signals and those originating outside the body (exteroception) (Tajadura-Jiménez and Tsakiris, 2014; Ainley et al., 2016; Badou and Tsakiris, 2017). Consistent with this assumption, several findings suggest that an emphasis on exteroceptive cues hinders interoceptive processing (Eshkevari et al., 2012; Crucianelli et al., 2016). Given that body image is a type of exteroception (see Dyrsdale and Tsakiris, 2021), relating to oneself and one’s body mainly via body image would make it more difficult to exercise and potentially enhance interoceptive abilities. In line with those studies, which found a positive correlation between low interoceptive sensibility and a negative body image, we assume that the dominance of a negative body image might interfere with existing interoceptive deficits. In other words, relating to our bodies “from the outside” impedes awareness of inner feeling states.

As shown in the previous subsection, negative body images prevail in incel forums, and are mutually maintained by social sanctions, for example by rating each other’s looks. If this coincides with what has been called “self-objectification” in psychological research (Fredrickson and Roberts, 1997), the perpetuation and identity-constituting function of a negative body image for incels may amount to a trait-like quality. In addition, several studies suggest a “significant relationship between autistic traits and indices of negative body image” (Longhurst, 2023, 9). In sum, findings on interoception difficulties in autistic people, on negative body image in autism, and research indicating a negative correlation between interoceptive awareness and self-objectification (Ainley and Tsakiris, 2013; Dimas et al., 2021) culminate in the hypothesis that a constantly evoked negative body image reduces the sense of agency (Koreki et al., 2022). Such a reduced sense of agency, in turn, speaks to a bodily felt dimension of hopelessness. In this sense, seeing ourselves through the eyes of others in a negative way tends to prevent us from being confident agents and raises doubts about what is possible for us at all, especially in terms of social connections. Importantly, the clustered findings suggest that autistic people are particularly vulnerable to the consolidation of social disconnection. In the next section, we examine how the peculiar dynamics of belonging and social discouragement in incel communities might lead to further aggravation of social disconnection from people outside the forums.

### 5.3 Mutually dismissive grieving communities

If we assume that the fundamental hopelessness of ever finding a romantic partner resembles the felt consequences of a “non-death” loss, we could also assume that they would entail similar ways of coping, namely striving for communal support in overcoming the situation. However, a number of studies (Maxwell et al., 2020; Helm et al., 2022; Regehr, 2022; Tietjen and Tirkkonen, 2023) suggest that incels “immunize” themselves instead against any prospect of alleviating their condition. Anyone who would provide encouragement or comfort lacks understanding and is considered a “normie,” which is irreconcilable with being a member of the incel community. In addition, incels who seek to change their situation by going on speed dating events, for instance, or paying for the services of “pick-up artists,” are accused of “red pill” behavior (believing they can improve their situation) and lying about their incel status. Similarly, incels who recount an everyday conversation with a woman are “put in their place” by their fellow community members for “bragging” (Kates, 2021; Tietjen and Tirkkonen, 2023).

Seeking help through therapy to cope with the felt loss of ever having intimate relationships does not seem viable for incels either. Studies show that many incels have undergone therapy (51.5%), but also that most did not find it helpful at all, or even that it made them feel worse (Speckhard and Ellenberg, 2022). In the online communities, therapy is discussed in a very negative light as a “scam” and form of social control (*ibid.*). In their view, therapists only “sell platitudes” that hide a demand to follow the norms of mainstream culture (*ibid.*). In essence, having therapy cannot change the things that they believe are “really wrong”: their looks, biologically determined laws of attractiveness, social norms, and women’s expectations. In this way, the deterministic worldview becomes interpersonally reinforced.

Instead of providing support and offering the possibility to make sense of one’s distress, the peculiar social dynamic upholds a state of hopelessness through repeated mutual dismissals and by simultaneously fostering the feeling of belonging. To be clear, this type of belonging centers around a purely negative group identity, which, contrary to other communities, is constituted by strong out-group demarcation, and does not entail complementary pride. Recalling the importance of social interactions for reorienting oneself after the loss of significant possibilities (Ratcliffe and Byrne, 2022), the social interaction patterns in incel communities provide a feeling of belonging at the expense of regaining hope.

Yet why, one might ask, do people need to resort to this type of belongingness at all? Besides the basic social glue facilitated by perceived similarity (Easterbrook and Vignoles, 2013), we argue that an important part of the answer lies in the media-specific features of online environments. We will elaborate on these features in the next section.

## 6 Toxic belonging in online communities *–* incel forums as detrimental affective scaffoldings

Several findings (Brosnan and Gavin, 2015; Roth and Gillis, 2015) suggest that people on the autism spectrum often prefer to form social bonds online instead of through “offline” encounters. The media specificities and the comparably lower “sensory load” of online interactions better accommodate the specific difficulties involved in multisensory integration and social cognition (Shalev et al., 2022). It is still commonly assumed that autistic people would not be interested in forming strong social connections, but recent studies have shown this assumption to be false (Jaswal and Akhtar, 2019). Rather, the difficulties experienced by autistic people in establishing social connections relate to predominant neurotypical environments (Syu and Lin, 2018; Krueger, 2021b; Williams D. J. et al., 2021; Williams G. L. et al., 2021).

In this section, we draw on so-called 4E approaches to online affectivity, that is, approaches that take embodied-affective phenomena as embedded, supported, or even co-constituted by the environment. Specifically, we seek to highlight how the media-specific features of particular online communities coalesce with the dimensions detailed in the previous sections (scientific determinism, body image dominance, and mutual dismissal). Thus, we wish to substantiate the claim that a specific sociomaterial environment can tap into particular vulnerabilities. Put differently, we claim that the incel forums may function as *detrimental affective scaffoldings*.

The notion of “affective scaffolding” has gained some traction in recent years in the so-called 4E approaches to affectivity. Building upon earlier work in developmental psychology (Vygotsky, 1978), “affective scaffolding” refers to the support provided by other people and the environment in bringing about affective states. This is usually couched in terms of “usage” in the sense that others or the material environment are deployed for eliciting, regulating, or maintaining affective states (Colombetti and Krueger, 2015). However, interactions in the incel forums show why this “user/resource model” (Slaby, 2016, 5) of scaffolding is too optimistic. In general, our affective lives are also shaped (Coninx and Stephan, 2021; Walter and Stephan, 2023), if not “invaded” (Slaby, 2016), by a broad range of scaffoldings.

In recent years, several authors have drawn attention to the ambivalent implications of becoming increasingly interwoven with online environments for our self-understanding, social identity, and eventually personal autonomy (Krueger and Osler, 2019; Alfano et al., 2021; Tremblay et al., 2021; Valentini, 2022). In the next section, we examine the extent to which the material and procedural properties of online environments might play a constitutive role in upholding detrimental social interactions.

### 6.1 Testimonial immunization

The first aspect concerns the multiplicity of online communities, which is unmatched in terms of accessibility in the “analogue” world. Finding like-minded people and social connections is simply much easier, particularly for those who have very special interests, beliefs, and preferences. However, the formation of echo chambers (encountering only similar-minded individuals) implies that this “freedom” of association is not universally beneficial (del Vicario et al., 2016; Nguyen, 2020; Cinelli et al., 2021). Rather, echo chamber dynamics may reinforce idiosyncratic beliefs that would be met with incredulous reactions elsewhere.

Furthermore, incel communities and their dynamics require an online infrastructure to exist. The sheer accessibility and availability of highly specific online communities facilitates their allure, also for those who face difficulties in neurotypical environments. This pull will only be stronger if these communities purport to be in possession of revelatory insights into fundamental laws of social existence. As suggested above, the effects of these digital infrastructures are not only confined to one’s belief systems and epistemic norms but can also become “embodied,” meaning that we literally incorporate them into our everyday lives.

### 6.2 Procedural integration of online structures

Internet-based interactions are pervasive in our everyday lives, and given the portability of many devices, this is tantamount to saying that we have integrated this informational infrastructure into our affective and cognitive lives (Krueger and Osler, 2019, pp. 223ff.). In fact, we could go as far as to say that we have embodied online devices via habituation of the multiple (regulatory) practices which are reliably enabled by these devices (Heersmink, 2015; Andrada et al., 2022). In pervading all domains of our everyday lives, the vast range of potential online interactions also entails the ubiquity of what Krueger and Osler (2019, 212) call the “hyper-social” aspect of “techno-social niches” (*ibid.*): “Others are often intensely present within the real-time dynamics of our regulative practices within these niches; and our practices, in turn, directly feed back into the regulative practices of others” (*ibid.*). In other words, other people and the roles they play in regulating our emotions are constantly present, and thus potentially shape our cognitive and, importantly, our affective processing.

The pervasiveness and the manifold interaction opportunities opened up by portable online devices also suggest that interactions that might at first sight appear to be confined to an “online space” will eventually also seep into other life contexts. For instance, while you are at a grocery store, a notification pops up on your phone indicating that several people have commented on your Reddit thread. Let us suppose that this is a subthread on incel-related topics, and simultaneously you are in the process of having to cope with an awkward social situation with an acquaintance that you suddenly bumped into in the store, and to whom you are secretly attracted. The new comments now confirm some inferences you have previously drawn from the psychological literature on sexual attraction. While there could have been many other possible ways to make sense of the frustrating interaction, the immediate intrusion of the Reddit debate pre-empts other ways of regulating emotions.

The fact that our regulatory practices can be pre-empted so easily also has to do with the high procedural transparency of online devices. We navigate apps, social media, and search engines without scrutinizing how they work, both in technical terms and in terms of their intentional or unintentional behavioral effects. That said, while transparency is not a prerequisite for external devices to become habitually entrenched (Smart et al., 2022), it does exert additional influence when it comes to shaping behavior, often leading us to let our guard down. In this sense, phenomenal transparency does not correspond with increased agency.

Research on situated affectivity, which emphasizes the influence of the situational context on several components of affective states, has borrowed the idea of “mindshaping” – originally developed to capture processes complementary to mindreading in social cognition (Zawidzki, 2013) – and applied it to affectivity. Whereas “(affective) mindshaping” denotes beneficial or detrimental effects of the influence of others on one’s affective life (Coninx and Stephan, 2021), the term “mind invasion” (Slaby, 2016) refers to intentional or unintentional malign effects. This can involve manipulating emotion-regulation capacities in multiple ways, affecting the choice of situations, modifying the situation itself, directing attention, prompting reappraisal, or influencing emotional expression (Gross, 1998). The ease of incorporating online devices will considerably amplify the impact on these capacities, particularly if online interactions serve as the sole or at least predominant medium for social connections.

### 6.3 Mind (and body) invasion online – tapping into vulnerabilities

In the previous subsections, we have highlighted the potential epistemic perils of online communities, their pervasiveness, and the procedural transparency of online devices. We conclude by tying up the loose ends and clarifying in what sense online interactions might invade one’s self-understanding. By “invasion,” we concur with Slaby (2016) and understand it as the regulation of one’s mental or bodily capacities against one’s own interests, which does not happen by coercion.

Thus far, we have specified the different dimensions along which incel communities engender hopelessness. Furthermore, we have shown that online environments have a specific capacity for disseminating beliefs, and occasionally socially and psychologically detrimental ones. Yet online environments have also become deeply integrated into our everyday lives and, importantly, into emotion regulation routines. This is not least due to the procedural transparency of the devices we use for navigating these environments. The latter aspect of pervasive but hardly conspicuous interwovenness has also been aptly termed “immaterial engagement” with the “cognitive ecology of the internet” (Clowes, 2019). Specifically, these pervasive (“ecological”) and at the same time tacit (“immaterial”) aspects of online environments contain a potentially “invasive” aspect, not only in terms of cognition but also in terms of affectivity (Valentini, 2022). In particular, online interactions mediated by smartphone use might attain a pervasive, encompassing, and sometimes dominating quality in regulating one’s emotions. Yet this is not only due to the procedural transparency of using smartphones, but also to the “seductive” qualities of the concrete user interfaces (Alfano et al., 2021). These seductive qualities result from the structural features of interaction opportunities, for instance in online forums or on social media platforms, as well as from highly individualized and algorithm-based recommendations (*ibid.*). In a detailed analysis of online political radicalization, Valentini (2022) has pointed out that the alternating dynamics of perceived interaction opportunities and constant prompts to interact with political extremist communities increase members’ engagement.

Given that these communities facilitate a certain worldview, taking into account the encompassing procedural and informational involvement, we would assume that the specific content thus conveyed might have an equally profound influence on one’s way of finding oneself in the world. More precisely, we would assume that the specific content with which one makes sense of the world, and the ways in which emotions are regulated within this infrastructure, leave a mark on one’s affective dispositions as a whole. Put differently, one’s affective dispositions and background feelings will most likely correlate with how one experiences the dominant and individually most significant social environment. Considering incel communities along these lines of pervasive and immersive engagement with an epistemically sealed-off ideology, we hypothesize a particular pull for persons who show “fitting” vulnerabilities.

In the previous sections, we have sought to establish that incel communities not only incite episodic emotions, but also radical hopelessness. Such a pervasive feeling is not restricted to the online interactions in these forums: rather, it stems from experiencing “non-death loss,” which affects one’s whole “being-in-the-world.” We argue that autistic people are particularly vulnerable to the dynamics that bring about and consolidate radical hopelessness in the incel forums. Autistic traits might cluster in ways that increase the likelihood of a lasting negative influence on one’s overall capacities for social attunement.

Of course, this is not to say that the higher likelihood of specific traits accumulating in autistic people is directly related to the adoption of an incel ideology. It is simply to say that these traits might pose difficulties when it comes to navigating neurotypical environments, and at the same time be highly compatible with forging social bonds online, and in incel forums in particular. Of course, true identification with and involvement in incel communities is also essentially contingent upon personality traits, many of which, and in the majority of cases, may override any tendencies to engage in the incel community.

However, if we assume that the longing for social connections manifests as a primary search for them in online interactions, perceived belonging to online communities may become a dominant feature for autistic people. In addition, a combination of sensory and communicative features in online forums might accommodate social cognition preferences; for example, the “excessive emotional empathy” recently identified in some cases of autism (Shalev et al., 2022) might be alleviated in the comparably less expressive information in online environments. Insofar as we can transfer the observations by Valentini (2022) on the intricate dynamics of feelings of agency and being constantly prompted or urged to engage in political extremist groups, a similar personal involvement occurs in incel forums. In particular, if online communities achieve a near monopoly on social connections, and in the absence or scarcity of corrective experiences, mind invasion processes are likely to be more effective. Further, as detailed above, “taking the black pill” promises a scientifically sound worldview that explains the experienced predicament of establishing social connections in a neurotypical environment, especially when it comes to finding romantic partners.

When the features of online incel communities detailed above (scientistic worldview, body image dominance, mutual dismissal) dominate one’s social, epistemic, and affective ways of relating to the world, this, most likely, also has an impact on one’s felt sense of significant possibilities *offline* – even more so, if the motivation to engage in these communities stems from trying to cope with the loss of social connections. In facilitating radical hopelessness, however, the allure of finding a sense of belonging ultimately exacerbates the pre-existing difficulties in establishing social connections.

## 7 Discussion: (re)situating phenomenological psychopathology

At the beginning of this article, we noted that phenomenological studies on autism have previously stressed the positive role of online environments in creating social bonds. We have called for a complementary, critical analysis of specific online environments which promise support and understanding, but which, at the same time, adversely affect one’s self-perception and felt possibilities of forming relationships. Previous empirical studies have also remarked that online interactions in incel communities entrench hopelessness. They have not, however, investigated what is meant by hopelessness or engaged in philosophical discussions of its experiential dimensions. By introducing phenomenological vocabulary into the discussion, we have argued that the forums consolidate the disorientation state of a “non-death” loss by diminishing felt life possibilities. Moreover, we suggest that the fundamental loss of hope and its implications for felt social possibilities tap into the particular vulnerabilities of people who already have difficulties in establishing social connections.

We have shown that in incel communities a certain existential feeling, a radical loss of hope, becomes the dominant affective disposition toward life. Since existential feelings are embodied feelings, radical hopelessness will also become embodied as individuals embrace the black-pill worldview. More precisely, we have hypothesized that by relating to oneself predominantly via body image, thereby thwarting interoceptive awareness, the incel ideology attains a bodily quality. It has been established that bodily feelings are crucial for social attunement (Fuchs and Koch, 2014; Grynberg and Pollatos, 2015; Ebisch et al., 2016; Arnold et al., 2019). Accordingly, we hypothesize that insofar as continuous and intensive involvement in incel communities leads to an overall alteration in how one feels about oneself, this will ultimately also affect how one perceives one’s capabilities to connect with others.

In line with canonical (Fanon, 2014) and more recently established critical phenomenological approaches to psychopathology (Zahavi and Loidolt, 2022), we therefore suggest that this alteration of existential feelings and consolidation of radical hopelessness indicates a “world-to-mind” influence on mental health. Yet while this might be understood as an oppressive relationship, we must attest that the feeling of belonging motivates one to participate in these communities. Thus, we acknowledge a rather bi-directional dynamic of maintaining hopelessness. Whereas traditional accounts of phenomenological and anthropological psychopathology have occasionally acknowledged the social dimension as an important factor in the formation of psychological distress, (von Gebsattel Freiherr, 1954; Jaspers, 1963; Binswanger, 1964; Blankenburg, 1983; Fanon, 2014), more fine-grained analyses of how the social context is involved have remained undeveloped. Hence, we have described the ways in which a particular set of social interactions conditioned by media-specific features may have a negative influence on people with specific vulnerabilities. More precisely, we have suggested that specific interaction patterns and a community-based worldview maintain, facilitate, and regulate existential feelings in such a way as to diminish significant life options and felt possibilities. Our approach builds on and responds to recent proposals to situate phenomenological psychopathology (Pienkos, 2020, 2022; Stanghellini, 2023) and provides an analysis complementary to other attempts to “re-contextualize the subject of phenomenological psychopathology” (Messas and Fernandez, 2022). We see our approach as an addition to work that has emphasized the importance of social interactions for people in at-risk states (Ritunnano et al., 2022). Although we do not claim that the interactions described here *necessarily* exacerbate the mental health situation of autistic people, we hypothesize that adopting an attitude of radical hopelessness will place considerable constraints on fostering social connections beyond the specific community.

More specifically, by showing how the combination of worldview, bodily self-relation, and ways of interaction leads to radical hopelessness, we have delineated how social and material factors might influence the mental health of those with specific vulnerabilities. The loss of radical hope characteristic of “blackpilling” might not necessarily amount to a major depressive disorder. However, since other forms of radical hopelessness also severely reduce felt life possibilities, we cannot exclude the fact that other more “fundamental” (Messas and Fernandez, 2022) phenomenological structures are also affected. For example, the felt loss of the aspiration to pursue life projects (Ratcliffe, 2013, 605) may indicate an alteration in the conative dimension and increase psychological distress. We do not deny that conditions such as depression or anxiety, both very common in incel communities, could also be linked to feelings of hopelessness. Here, however, we have focused on non-neurotypical experiences because it is even less clear how they relate to hopelessness, thus pointing to contextual factors. That said, we do not base our hypotheses on factual diagnoses but focus more broadly on autism-related traits commonly recognized in the research literature. Moreover, we do not claim that all autistic people are inclined to join incel communities. Future research should investigate the precise trait factors that increase the likelihood that individuals with difficulties in forming social connections will adopt an incel worldview.

Looking ahead, we see great potential in further investigating the impact of socially constituted body image dimensions on affective dispositions and interoceptive capacities. This would also be fruitful for integrating phenomenological approaches to the lived body with recent advances in critical phenomenology that focus explicitly on the social formation of lived experience.

In highlighting the occasionally intricate dynamics of belonging, we stress the importance of lowering the thresholds for autistic people to navigate neurotypical environments, namely, to foster “atmospheres of inclusion” (Krueger, 2021a), and to combat bullying within mainstream neurotypical environments. This also amounts to not taking supposed preferences (*viz.* “relative preferences”) for online interactions as an easy excuse for not providing social contexts better suited to neurodiverse persons, since, as we hope to have clarified, online interactions are not *per se* only beneficial.

## 8 Conclusion

We have analyzed the experiential connections between autism and hopelessness in online forums for involuntary celibates (incels). Rather than taking for granted what is meant by hopelessness, we have distinguished between its intentional and more profound forms, and have asked how they are present in the experience of incels. In the case of autism, the fundamental loss of hope is conditioned by vulnerabilities and difficulties in establishing social connections. We have asked how these feelings of hopelessness are fostered in online interactions, and how these interactions function as detrimental affective scaffoldings. We have focused on three dimensions of these interactions: scientific-deterministic explaining, the negative shaping of body image, and the tendency toward mutual dismissal. Participation in these communities is motivated by the wish to belong, but instead of finding solidarity and support for overcoming the situation, hopelessness is maintained through the bi-directional dynamic and media-specific features of the forums. We argue that the alteration of existential feelings and consolidation of radical hopelessness indicates a “world-to-mind” influence on mental health. In this way, our approach contributes to the debate and the ongoing task of (re)situating phenomenological psychopathology – also in contemporary online environments.

## Data availability statement

The original contributions presented in the study are included in the article/supplementary material, further inquiries can be directed to the corresponding author.

## Author contributions

Both authors listed have made an equally substantial, direct, and intellectual contribution to the work, and approved it for publication.

## Footnotes

^3^This dynamic interrelation has already been noted in Merleau-Ponty’s (2011, 157ff., own translation) distinction between the "infrastructure" (implicit) and the "superstructure" (explicit) of the body.
